# Coexisting salps exhibit distinct feeding selectivity on microorganisms in the North Pacific Subtropical Gyre

**DOI:** 10.21203/rs.3.rs-8605244/v1

**Published:** 2026-01-27

**Authors:** Anne W. Thompson, Kylee Lamberson, Kelly R. Sutherland

**Affiliations:** Portland State University, Department of Biology, PO Box 751, Portland, OR, 97201; Portland State University, Department of Chemistry, Portland, OR, 97201; Oregon Institute of Marine Biology, University of Oregon, Eugene, OR 97403

**Keywords:** *Crocosphaera*, nitrogen fixation, microbial ecology, *Prochlorococcus*, *Synechococcus*, SAR11, mixotrophy, grazing, salps, pelagic tunicates, North Pacific Subtropical Gyre, microbial loop

## Abstract

Mortality mechanisms play an important role in how oceanic microorganisms contribute to global biogeochemical cycles. Salps are widespread pelagic tunicates known to remove phytoplankton from coastal and high-latitude waters, but their interaction with microorganisms in the vast tropical and subtropical gyres is not well quantified. Using quantitative measurements of six major marine microorganisms in the guts of six distinct but co-occurring salp species from the North Pacific Subtropical Gyre, we examined the impact and dynamics of salp feeding on marine microorganisms in a vast open ocean region. All salps preferentially removed prey greater than 1 μm in diameter, including marine *Synechococcus*, diatoms, *Crocosphaera*, and *Chrysochromulina*, while the smaller *Prochlorococcus* and SAR11 were not a major source of prey biomass. We also found that salp feeding varied between salp taxa with some salp guts dominated by both *Crocosphaera* and *Chrysochromulina* while others were dominated by *Crocosphaera* alone. Together, these results suggest that salp impacts are not uniform across taxa and their patterns of selective feeding among marine microbes requires consideration of species-specific feeding strategies and environmental context. Further, this work suggests that the mortality pressure of salp feeding on marine microorganisms may shape microbial community structure and that this pressure varies with the diversity and dynamics of macrozooplankton predators.

## Introduction

Microorganisms of the ocean’s vast subtropical gyres play essential roles in global biogeochemical cycles. Photosynthetic microorganisms in the open ocean gyres contribute to primary production on global scales (Field et al. 1998). Nitrogen fixing microorganisms make fixed nitrogen widely available to other members of the microbial community (Goebel et al. 2007). Protists propagate carbon from bacterial prey to higher trophic levels (Levinsen et al. 2000). And, the numerically dominant heterotrophic bacteria recycle complex carbon compounds (Azam 1998). Together these distinct yet interconnected metabolisms affect the global carbon cycle and support pelagic ecosystems (Azam and Malfatti 2007).

The mortality of open ocean microorganisms is one important, but understudied, factor that drives how these diverse microbial communities contribute to global processes. How a marine microorganism dies affects the carbon compounds released to the environment (Ma et al. 2018), the availability of the cell’s nutrient and energy for transfer to higher trophic levels, and the direct and/or indirect interactions between remaining cells (Follett et al. 2022). While many diverse mortality sources of marine microorganisms have been identified, including viruses (Arsenieff et al. 2022; Carlson et al. 2022), protists (Li et al. 2022; 2021; Landry et al. 2023), and macrozooplankton (Almeda et al. 2018; Sutherland et al. 2010; Stukel et al. 2021), the details of these feeding interactions are not well known. How distinct mortality processes impact microbial ecology and evolution, control feeding rates, respond to the environment, and vary across different types of prey and predators remain to be discovered (Kerkar et al. 2024).

Salps are globally abundant macroplanktonic filter feeders that selectively feed on marine microorganisms (Luo et al. 2022; 2020; Fender et al. 2022; 2025; Stukel et al. 2021). Across the 41 species known worldwide (Damian-Serrano et al. 2023), salps differ in their feeding rates and selectivity due to differences in behaviors, morphology, and mucous mesh pore sizes (Madin and Kremer 1995; Madin and Cetta 1984; Fender et al. 2022; 2025). However, most insight into salp selective feeding has been achieved in temperate or polar systems and with emphasis on the eukaryotic prey prevalent in those systems. Thus, there remains a gap in quantifying the feeding rates and selection of different salps from tropical and subtropical systems on smaller prey, especially abundant marine prokaryotes.

In this study, we compared the feeding of five different co-occurring salp types on six different marine microorganisms that included heterotrophic bacteria, picocyanobacteria, diazotrophs, and eukaryotic phytoplankton. We found that all salps fed preferentially on larger phytoplankton prey (>1 μm) over smaller phytoplankton and heterotrophic bacteria (<1 μm). However, there were clear differences between the salp species in which open ocean microorganisms made up the bulk of their prey biomass. We also estimated the impact of salp feeding on microbial communities from the open ocean gyres by calculating clearance and prey retention rates from the quantitative counts of prey in each salp gut in combination of existing estimates of salp abundance during steady-state and bloom conditions in the North Pacific Subtropical Gyre (NPSG). These results demonstrate that salps vary between species in their microbial prey selection, can exert a strong influence on larger members of the microbial communities (> 1 μm), and consume the ocean’s smallest cells (<1 μm) at low rates even under bloom conditions.

## Materials and Methods

### Salp and seawater collections

Samples were collected 3 nautical miles offshore of Kona, Hawai’i (19.710746 N, 22.75 W), within the North Pacific Subtropical Gyre (NPSG). Salps and seawater were accessed by a recreational dive boat using blue-water SCUBA techniques (Haddock and Heine 2005) within 2 hours of sunset in approximately the top 25 meters of the water column. Divers slowly approached and enclosed salps in 1-liter jars to prevent the salps from ejecting their nets, evacuating guts, or stopping feeding. Immediately after capture, a 50 mL sample of the seawater prey field was collected with a syringe in the area near the captured salp. Both the seawater prey field samples and captured salps were returned to the surface for dissection and preservation within 15 minutes of collection. Numbers of specimens for each salp taxa are as follows: *Brooksia rostrata* (5), *Cyclosalpa affinis* (19), *Cyclosalpa quadriluminus* (17), *Pegea confoederata* (15), *Salpa maxima* (10), *Iasis cylindrica* (8), seawater (12). We reported *P.confoederata* clearance rates and selection patterns in a previous work (Thompson et al. 2024). In this current study, we bring in all other salps listed above for comparison.

### Salp gut and fecal pellet collection and DNA extraction

Salp gut dissections were performed as previously described (Thompson et al. 2024). Briefly, dissections were performed within 15 minutes of collection. Any intact fecal pellets at the bottom of the collecting jar were transferred by wide-bore pipette to a sterile bead-beater tube pre-loaded with 100 μl DNA/RNA Shield (Zymo Research, Irvine, CA). Salps were gently poured from the collection jars onto a mesh sieve and gently rinsed three times with filtered (0.2 μm) seawater. Dissection scissors were sterilized in ethanol and treated with DNA AWAY^™^ (ThermoFisher Scientific, Waltham, MA) before cutting the gut out of the animal and placing it in a sterile bead-beater tube pre-loaded with 100 μl DNA/RNA Shield. Gut volumes were estimated, based on their shape and diameter. DNA was extracted from seawater filters, salp guts, and fecal pellets using the DNeasy Plant Tissue Mini kit (Qiagen, Hilden, Germany), which was chosen for effective lysis of prokaryotic phytoplankton.

### Quantitative PCR for six marine microorganisms

Quantitative polymerase chain reaction (qPCR) was used to measure the abundance of six different microbial prey types in the DNA from dissected salp guts and the seawater prey field (50 mL volumes filtered on 0.2 μm pore-sized filters). As previously published for *P. confoederata* (Thompson et al. 2024), we targeted *Prochlorococcus* MIT9312, SAR11, marine *Synechococcus* (Clade 2), *Crocosphaera*, the diatom *Thalassiosira sp*., and the haptophyte (and known *Prochlorococcus* predator) *Chrysochromulina*, with well-established qPCR assays. All these microbial preys are known to be present in the NPSG surface waters near our field site. We corrected gene copies per mL detected by qPCR to cells per mL based on copy number per cell, which was 1 for all bacterial assays, 2 for *Chrysochromulina* (Li et al. 2022), and 1 for the diatom (with the caveat that diatom 18S rRNA gene copy number per cell can vary substantially across taxa, as described in Gong and Marchetti 2019 (Gong and Marchetti 2019).

We performed the qPCRs with the Power SYBR^™^ Green PCR Master Mix (Applied Biosystems, Waltham, MA) or the TaqMan^™^ Universal Master Mix (Thermo Fisher Scientific, Waltham, MA), depending on the published assay protocols, detailed previously (Thompson et al. 2024). To reduce PCR inhibition, we diluted (1:5) the DNA with nuclease-free water and added 1 μL of 20 mg/mL recombinant albumin (RA) (New England Biolabs, Beverley, MA) to each qPCR reaction to a 1 mg/mL final concentration. Standard curves for each assay were created between 10^0^ and 10^6^ gene copies using gBlocks synthetic oligonucleotides (Integrated DNA Technologies, Coralville, IA). All reactions were run in triplicates on a ViiA 7 Real-time PCR system (Thermo Fisher Scientific, Waltham, MA) in 20 μl volume, with a melt curve analysis at the end of the cycles to confirm that only one amplicon was produced.

### Microbial biomass calculations

We estimated the biomass contributed by each microbial prey type. Microbial population biomass (pg C L^−1^) was calculated by multiplying previously published per-cell carbon quotas by the number of cells per volume (measured in this study via flow cytometry or qPCR). While there are per cell carbon quotas published for taxa similar to *Chrysochromulina* and *Thalassiosira* (Ishiwata et al. 2013; Menden-Deuer and Lessard 2000; Ribalet et al. 2019), we used biomass estimates for these two cell types based on their estimated spherical diameters for pigmented picoeukaryotes in the North Pacific Subtropical Gyre, as published previously (Ribalet et al. 2019).

### Ingestion and clearance rate calculations

We calculated ingestion and clearance rates on the different microbial prey using approaches and equations published previously as the “gut particle content method” (Madin and Kremer 1995). Gut passage times for the salps we sampled were estimated from data provided previously (Madin and Cetta 1984) that related salp size to gut passage time, inputting our salp measurements.

### Impact on microbial prey populations calculations

To estimate the impact of salps on marine microbial populations in subtropical regions we calculated the percentage of each microbial prey population in a cubic meter of seawater that would be removed by salps each day. For these calculations, we followed an approach previously used to estimate the impact of ctenophore feeding on zooplankton prey (Irvine et al. 2025). For our study, we used our average salp clearance rates (i.e., the mean clearance rates of all salp species). Next, to calculate the percentage of microbial prey populations in each cubic meter of seawater removed by salps daily, we used measured salp abundances in the subtropical gyre from Station ALOHA (Steinberg et al. 2008) near our field site, where the average number of salps is about 1 salp m^−3^, to estimate the impact during steady state conditions and estimated salp abundances from other subtropical gyres where salps bloom to > 10 salps m^−3^ (Stone and Steinberg 2014; Ishak et al. 2022)

## Results

### Seawater prey field

Measurements from qPCR of different free-living microorganisms (“microbial prey”) in the seawater (i.e., prey field) from which the salps were collected showed that *Prochlorococcus* ecotype MIT9312 and SAR11 were the most abundant at nearly 100,000 cells per mL of seawater. The numerical dominance of these two cell types is consistent with prior measurements in the North Pacific Subtropical Gyre (Karl and Church 2017).

All other prey ranged between 10-1000 cells per mL, including the cyanobacterial diazotroph *Crocosphaera*, mixotrophic haptophyte *Chrysochromulina*, diatoms, and the picocyanobacterium *Synechococcus*. These abundances were also consistent with previous measurements of *Synechococcus* (Karl and Church 2017), *Crocosphaera* (Dugenne et al. 2020), diatoms (Armbrust 2009), and *Chrysochromulina* (Li et al. 2022) from the region.

### Salp taxa collected

Multiple taxa of salps were encountered and collected during dives at the sample station. Salp taxa included *P. confoederata*, *B. rostrata*, *C. affinis*, *C. quadriluminus*, *I. cylindrica*, and *S. maxima*. For collection, we targeted salp taxa that could fit into our 1-liter collection jars and were abundant enough to gather replicates for robust comparison with other taxa. Thus, these salp species are an indication of what was present in the system, however the collection design was not meant to quantify their absolute or relative abundances.

### Microbial prey counts in guts, fecal pellets, and seawater

**Figure 1.**
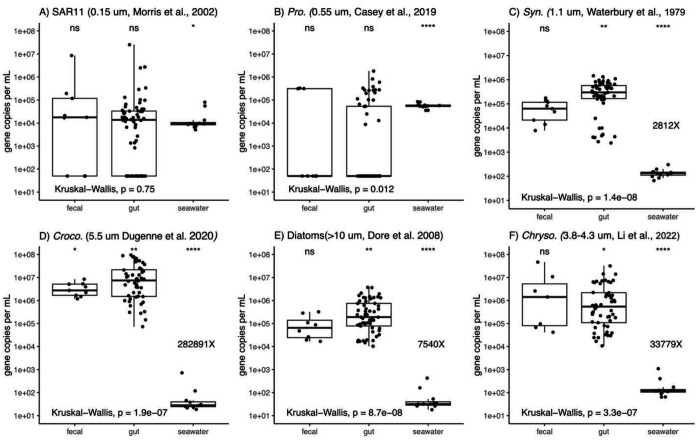
Comparison of microbial prey taxa in salp fecal pellets, salp guts, and the surrounding seawater. References to microbial size accompany each panel. Comparisons of the median for each sample type were performed with Kruskal-Wallis. For microbial prey with significant (p-value < 0.01) differences between sample types, the fold change between the median of the seawater and salp gut are indicated. Abbreviations: Pro. (*Prochlorococcus*), Syn. (*Synechococcus*), *Croco*. (*Crocosphaera*), *Chryso*. (*Chrysochromulina*), not significant (ns), p-value (p).

Next, we compared the gene copies per volume of each microbial prey taxa across the three sample types (seawater, salp guts, and salp fecal pellets) (Figure 1). Both SAR11 and *Prochlorococcus* gene copies per volume were the same across all sample types (*p-value* > 0.01, Kruskal-Wallis), with no significant enrichment in fecal pellets or salp guts relative to the background seawater (Figure 1A–B).

In contrast to *Prochlorococcus* and SAR11, the other prey types were highly enriched in salps guts and fecal pellets relative to the background seawater (p<0.01, Kruskal-Wallis). *Synechococcus* and diatoms were more than 1,000-fold enriched in the salp guts and fecal pellets relative to the seawater (Figure 1C+E). *Chrysochromulina* was over 33,000-fold enriched in the fecal pellet and guts relative the seawater (Figure 1F). *Crocosphaera* was the most enriched, at over 280,000-fold more enriched in the salp guts and fecal pellets relative to the seawater (Figure 1D).

### Microbial prey counts in guts varies by salp taxa

**Figure 2.**
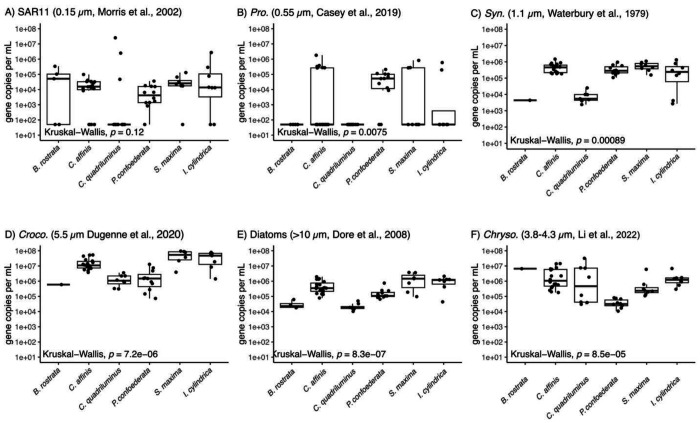
Comparison of how different salp species retain each microbial prey in their gut. References to microbial size accompany each panel. Comparisons of the median for each sample type were performed with Kruskal-Wallis. Abbreviations: Pro. (*Prochlorococcus*), Syn. (*Synechococcus*), *Croco*. (*Crocosphaera*), *Chryso*. (*Chrysochromulina*), p-value (p).

Next, we compared the six salp types in number of each microbial taxa they retained, per their gut volume (Figure 2). For each prey type, salps *S. maxima*, *I. cylindrica*, *P. confederata*, and *C. affinis* contained about the same number of prey cells per gut volume. However, *C. quadriluminus* presented a different pattern. This species mostly contained fewer larger phytoplankton prey cells compared to the other salp species, often with a difference of two orders of magnitude.

As it was not possible to exactly quantify the gut volume of each salps, we explored whether this difference could be due to the estimated size of the gut. With that, even a 10-fold error in the determination of gut volume would still leave a difference between *C. quadriluminus* and the other taxa. We also noted that a subset of the *C. quadriluminus* samples contained measurable counts of SAR11, exceeding the other samples by multiple orders of magnitude (Figure 2A–B).

**Figure 3.**
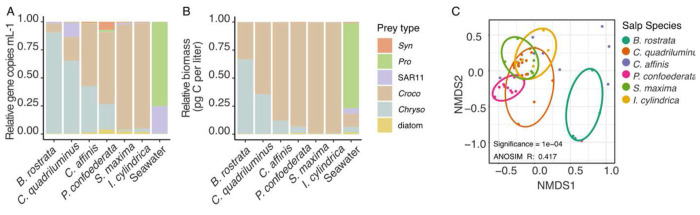
Comparison of different salp species in their A) relative abundance of the different microbial prey they contain and B) the estimated biomass contributed by each prey type within each salp type. C) NMDS comparing salps based on their prey content. Ellipses encompassing data from each salp type are drawn by eye. Whether salp species was significant is driving patterns on the NMDS was tested by ANOSIM with display of significance and strength of the effect (R).

The next step we took in comparing salps was to assess the relative abundance of each prey type in the different salp species (Figure 3A). We undertook this analysis, using means of the abundance of each prey shown in Figure 2, in order to compare the relative composition of prey in each salp, rather than the absolute abundance of prey. The seawater prey field microbial community was distinct from all salps and the different salp types varied in the relative abundance of the different prey. *C. quadriluminus* was particularly distinct from the other salps in that it contained relatively more SAR11 than the other salps. *B. rostrata* and *C. quadriluminis* guts were numerically dominated by *Chrysochromulina* cells. *P. confoederata* and *C. affinis* were similar to each other in the representation of *Crocosphaera* and *Chrysochromulina* cells in their guts. This pattern contrasted with *S. maxima* and *I. cylindrica* guts, which were numerically dominated by *Crocosphaera* cells. Non-metric multidimensional scaling (NMDS) analysis confirmed that the prey content of the salp taxa were different (Figure 3C). While the differentiation between the salps based on their prey taxa was only moderately strong (ANOSIM R ~0.4), the salp taxa were significantly different by their prey composition (ANOSIM significance < 0.01). *B. rostrata* was most distinct from the other salps. There were also distinct differences between *P. confoederata* and *I. cylindrica*.

### Relative contribution of individual prey taxa to overall prey biomass

When we looked at which prey contributed the most biomass to salp prey content we found that *Crocosphaera* contributed the most biomass to all salp types, except for *B. rostrata*. In contrast, *B. rostrata* contained more *Chrysochromulina* than *Crocosphaera*. Overall, for all salps, *Synechococcus*, *Prochlorococcus*, SAR11, and diatoms contributed very little towards the prey biomass, in contrast to their contributions to the microbial community biomass in the seawater.

### Calculated ingestion and clearance rates

Calculated ingestion and clearance rates of salps varied across the six different microbial prey types. Ingestion rates of *Crocosphaera* were highest and ingestion rates of *Prochlorococcus* and SAR11 were lowest. Clearance rates were also highest for *Crocosphaera* at around 10,000 mL per salp per day (or 10-liters per salp per day) and lowest for *Prochlorococcus* and SAR11. The calculated clearance rates were similar to our previous direct measurements of clearance rates in bottle incubations with *P. confoederata* (Thompson et al. 2024).

### Impact of salp feeding on phytoplankton populations

**Figure 4.**
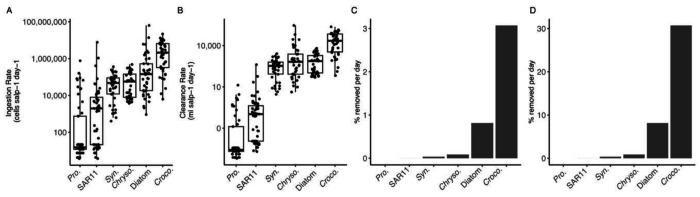
Comparison of the impact of salp feeding across microbial prey taxa. A) Comparison of ingestion rate of each microbial prey by cell. B) Clearance rate of each microbial prey type. C) estimated percent of microbial prey cells, by microbial taxa, consumed in one cubic meter per day by salps (estimated abundance at 1 salp per cubic meter, from Steinberg et al. (2008) at Station ALOHA), and D) estimate with salps at “bloom” conditions, a conservative estimate of 10 salps per cubic meter (Stone and Steinberg 2014; Ishak et al. 2022). Abbreviations: Pro. (*Prochlorococcus*), Syn. (*Synechococcus*), *Croco*. (*Crocosphaera*), *Chryso*. (*Chrysochromulina*).

To understand the impact of salps on microbial communities of the NPSG, we calculated the percent of the phytoplankton population in a cubic meter that could be removed by salps each day under non-bloom “steady state” conditions (Figure 4C) and “bloom” conditions (Figure 4D) for salp abundances. For non-bloom estimates, we used Steinberg et al. (2008) salp abundances at Station ALOHA at one salp per m^3^. Under non-bloom conditions, we estimated that less than 0.1% of *Prochlorococcus*, SAR11, diatoms, and *Synechococcus* populations would be removed per day. For *Chrysochromulina*, we estimated that under non-bloom conditions salps could remove about 0.8% of the population each day. *Crocosphaera* removal was the highest, with salps estimated to remove about 3% of the population each day under a non-bloom scenario.

We also estimated the salp feeding impact on each prey under a salp bloom scenario, where we estimated conservatively at >10 salps m^−3^ based on studies at the Bermuda Atlantic Time Series (BATS) (Stone and Steinberg 2014) (Figure 4D). Under bloom conditions, we found that up to 30% of the *Crocosphaera* and up to almost 10% of the *Chrysochromulina* populations could be removed. The other prey taxa were still removed at very low levels.

## Discussion

To expand understanding of how predation interactions contribute to microbial community composition and biogeochemical function of the oligotrophic open ocean, this study examined the feeding of six coexisting species of salps on a diverse set of microorganisms in the North Pacific Subtropical Gyre (NPSG). While feeding selectivity of salps on microbial populations is well known for the largest microbial taxa (such as eukaryotic phytoplankton) (Dubischar and Bathmann 1997; Fender et al. 2022), especially in temperate and polar seas, much less is known about the impact of salps on picoplankton (< 2-3 μm in diameter) and whether salp species differ in their ability to shift specific picoplankton functional groups through selective feeding.

### Salps feed on picoplankton in addition to larger prey

One strong signal of this study is that diverse open ocean salps consistently retained a range of prey sizes, from the largest cells we studied, *Crocosphaera* (5.5 μm in diameter (Dugenne et al. 2020)), down to cells in the range of picoplankton (including the picocyanobacterium *Synechococcus*, ~1 μm diameter). Previous microscopic studies that did not identify the taxonomy of prey cells (Silver and Bruland 1981; Hiebert, Thompson, et al. 2025) and experiments with inert microspheres (Kremer and Madin 1992) suggested that salps can retain cells at the upper range of picoplankton (with picoplankton defined as 0.2-2 μm in diameter). Our qPCR data confirms this with the presence of *Synechococcus* in salp guts. Together these studies also support previous work which quantified salp mesh sizes and predicted retention of picoplankton sized prey, including those at about 1 μm in diameter (Sutherland et al. 2010).

Removal of *Synechococcus* suggests that other picoplankton in this size range may also be likely prey of salps. If salps are numerous enough, for example during a bloom scenario, the removal of this abundant category of cells by salps could have moderate implications to the level of biogeochemical cycling and primary productivity carried out by picoplankton in the oligotrophic open ocean as picoplankton in the NPSG play vital ecosystem roles. For example, predatory flagellates from the class Chrysophyceae can be as small as 2 μm (Li et al. 2022), and so they could also be removed by salps leading to reduced predation on bacteria in cases where salps are abundant. Similarly, stable isotope labeling demonstrates that picophytoplankton make up more than 60% of the microbial phytoplankton biomass and fixed more CO_2_ than picocyanobacteria in an oligotrophic subtropical gyre (Duhamel et al. 2019), thus removal by salps could slow this contribution to the carbon cycle. Future work with more qPCR assays that target a broader suite of picoplankton (especially unknown are the diverse heterotrophic picoplankton) will illuminate these additional biogeochemical and community structure consequences of grazing by macroplankton.

### Diazotroph Crocosphaera and mixotroph Chrysochromulina removed at high rates relative to diatoms

Though the smallest cells (< 1 μm in diameter) including SAR11 (0.15 μm, minimu diameter) and *Prochlorococcus* (0.55 μm) were not retained by salps, cells equal to or larger than 1 μm were captured at elevated rates compared to the background seawater across all salp species. Consistent with previous work (Thompson et al. 2023; Dadon-Pilosof et al. 2019; Hiebert, Aasjord, et al. 2025), particle capture was not based solely on prey cell size, suggesting that other factors like cell shape, nutritional quality, surface properties, or digestibility may influence selection. Specifically, *Crocosphaera* (5.5 μm) and *Chrysochromulina* (3.8-4.3 μm) numerically dominated salp guts and fecal pellets and were captured at higher rates than diatoms (> 10 μm), even though diatoms were available in similar concentrations in the background seawater. In the NPSG, while not the largest phytoplankton, the diazotroph *Crocosphaera* comprises a large portion of the nanoplankton (~30%) and is grazed by dinoflagellates and ciliates (Dugenne et al. 2020), which suggests a density dependent grazing relationship that links primary and secondary production and exerts control on overall N_2_ fixation in the study region. The nanoflagellate *Chrysochromulina* was another prominent prey item in salp guts. *Chrysochromulina* plays a unique role as the most abundant mixotroph at station ALOHA, capable of exerting topdown control of *Prochlorococcus* (Li et al. 2022). The removal of these cells by salps therefore has the potential to release grazing pressure on *Prochlorococcus* and other bacterial prey. Diatoms were also captured at relatively lower rates than *Crocosphaera* even though their background concentrations were similar to *Chrysochromulina*. However, it is important to note that the abundances we measured are a snapshot of what is known to be a dynamic phytoplankton community, as the NPSG is dynamic in space and time with recurrent summer phytoplankton blooms as well as finer scale spatial and temporal shifts (Tucker et al. 2024; Dore et al. 2008). Salp grazing impact should be expected to vary as the background community shifts exerting a measurable but dynamic predation pressure on the numerically abundant small eukaryotic phytoplankton that are core contributors to primary production and nitrogen fixation in the open ocean.

### Salps impact on microbial biomass and counts in the open ocean depends on population density

Based on an average steady state estimate of salp abundance in the sampling region (Steinberg et al. 2008), we estimated low impact of salps on the cell counts (< 1%) of microorganisms in each cubic meter of water from the surface ocean (Figure 4C), with the exception of *Crocosphaera* (~3%). These estimates are consistent with previous measures of the low impact of salps on microbial biomass in waters spanning the subantarctic to subtropical front (Stukel et al. 2021). The low impact of salps on open ocean microbial communities from these two studies bolsters arguments that viruses and protist predators may be the more important microbial predators in the surface ocean under steady state conditions. For example, Stukel et al. (2021) quantified protist and salp mortality at the same time and found that protists exhibited higher grazing rates as a whole.

However, salps are notoriously patchy and can form dense blooms, reaching up to 100 salps per cubic meter, or more, in oligotrophic environments (Stone and Steinberg 2014). Further, multiple salp species frequently co-occur in tropical oceans as we found in the current study, compounding the collective grazing impact. Finally, current estimates of salp abundance in the open ocean are likely underestimates given their tendency to break apart in nets (Henschke et al. 2016). Emerging *in situ* image-based techniques may help to better constrain the true abundance and grazing impact of salps (e.g. (Greer et al. 2020)). During blooms, fast-sinking salp fecal pellets transport carbon to depth and represent another ecological impact that hinges on the identity of removed cells (Madin 1982). Fecal pellets and salp gut contents contained a comparable microbial assemblage and cell abundances (Figure 1), which suggests that salp grazing contributes to both carbon removal and export flux. The available evidence suggests that, on average, salps exert a low impact on microbial communities, but under bloom conditions, salps have the potential to substantially impact microbial biomass, abundances and vertical export of carbon from surface waters.

### Co-occurring salps vary in their feeding preferences

Salps vary in their morphology, movement and behavior leading to our hypothesis that different salps may have different feeding preferences on their prey (Figure 2). We opportunistically collected multiple species at our study site, representing the broad diversity of salp species and colony architectures (Damian-Serrano et al. 2023). While all salps feed by pumping water through their barrel-shaped bodies and capturing particles on a fine mucous mesh, different salp species vary in size, pulsation rates and depth of contraction. These differences drive overall fluid processing, i.e., clearance rate (Sutherland and Madin 2010), but may be less important for particle selection. On the other hand, differences in mesh size or mesh biochemical properties could account for differences in selection by different species as we observed (Figure 2–3). Salp mesh openings are around 1 μm in diameter and have been shown to vary across species (Bone et al. 2003) and increase with body size within a species (Sutherland et al. 2010). At small size scales of mesh openings, even minute differences in dimensions or fluid motion are expected to influence how particles interact with the mesh (Thompson et al. 2023). Stickiness of the mesh could also drive which individual particles adhere and are ultimately ingested. These features could help explain why *C. quadriluminus* and *B. rostrata* had guts dominated by *Chrysochromulina* while *S. maxima* and *I. cylindrica* had guts dominated by *Crocosphaera* (Figure 3). *C. quadriluminus* stood out in particular as it consumed SAR11 at higher rates than other salp species, even though these cells represented an inconsequential carbon contribution.

Differences in salp morphology are frequently linked to different swimming behaviors, which could influence the microbial prey they contain. Species with long linear chain architectures, like *S. maxima*, are known to vertically migrate whereas species with less streamlined transverse chain architectures, like *P. confoederata*, are restricted to surface waters (Madin et al 1996). Other species, like *C. affinis*, undertake only shallow migrations on the order of tens of meters. Salps that migrate deeply (100 m or more) may encounter a different microbial assemblage and therefore consume different prey from primarily surface associated salps. Considering that it takes several hours for salps to completely clear their guts (Madin & Cetta 1984), species migrating from deeper waters like *S. maxima* could contain prey representing a background assemblage different from the seawater we sampled surrounding the salp. Further work could elucidate how distinct grazing preferences among co-occurring salp species mediate impacts on the microbial community.

## Conclusion

In conclusion, this study indicates that salps exert complex effects on open-ocean microbial communities through their selective feeding. Under non-bloom conditions, their impact appears minimal; however, during bloom events salps can consume large quantities of picoplankton prey (~1 μm in diameter and greater) within a single day, at rates potentially sufficient to influence biogeochemical cycling. When feeding, salps exploit a broad spectrum of available microbial cells, including cells at the upper size range of picophytoplankton, yet they largely pass over the smallest and most abundant microbial taxa, such as SAR11 and *Prochlorococcus*. A key contribution of this work is that salp impacts are not uniform across taxa: species such as *C. quadriluminus* exhibit divergent feeding patterns and selectivity, underscoring that salps cannot be treated as a functionally homogeneous group. Collectively, these findings highlight the need to consider both environmental context and species-specific feeding strategies when evaluating the role of salps in structuring and regulating the biogeochemical processes of microbial communities.

## Data Availability

The datasets generated during the current study will be available in the Biological and Chemical Oceanography Data Management Office (BCO DMO) repository: https://www.bco-dmo.org/project/929558
